# Prognostic value of microRNA-125a/b family in patients with gastric cancer: a meta-analysis 

**Published:** 2021

**Authors:** Nasrin Amiri-Dashatan, Mehdi Koushki, Mohsen Naghi –Zadeh, Mohammad Reza Razzaghi, Hamid Mohaghegh Shalmani

**Affiliations:** 1 *Proteomics Research Center, Shahid Beheshti University of Medical Sciences, Tehran, Iran*; 2 *Department of Clinical Biochemistry, School of Medicine, Zanjan University of Medical Sciences, Zanjan, Iran*; 3 *Department of Clinical Biochemistry, Faculty of Medicine, Tehran University of Medical Sciences, Tehran, Iran*; 4 * Laser Application in Medical Sciences Research Center, Shahid Beheshti University of Medical Sciences, Tehran, Iran*; 5 * Gastroenterology and Liver Diseases Research Center, Research Institute for Gastroenterology and Liver Diseases, Shahid Beheshti University of Medical Sciences, Tehran, Iran*

**Keywords:** Gastric cancer, Meta-analysis, Mir-125 A, Mir-125b, Prognosis

## Abstract

**Aim:**

This meta-analysis was designed to reassess the prognostic and clinicopathologic values of the microRNA-125 family in GC patients.

**Background:**

The miR-125 family (including miR-125a, miR-125b) has been reported as being pivotal prognostic biomarkers of gastric cancer (GC). However, there is controversy about the role of the miR-125 family in predicting the progression of GC.

**Methods:**

The miR-125 family (including miR-125a, miR-125b) has been reported as being pivotal prognostic biomarkers of gastric cancer (GC). However, there is controversy about the role of the miR-125 family in predicting the progression of GC.

**Results:**

The electronic databases of PubMed, ISI Web of Science, Scopus, and Cochrane Library were systematically searched for relevant studies. Overall survival (OS) rate as the primary outcome from each study was extracted. The overall hazard ratio (HR or survival rate in patients with GC) and odds ratio (OR) with 95% confidence interval (CI) was calculated to evaluate the association between miR-125 family expression and prognosis and susceptibility to gastric cancer. The quality of evidence was evaluated using the Newcastle-Ottava Scale (NOS). The extracted data was combined based on the random-effects model.

**Conclusion:**

The low expression of miR-125 family predicts poor OS in GC patients. Thus, the miR-125 family may be helpful as a potential biomarker for the prognosis of gastric cancer.

## Introduction

 Cancer is a critical threat for human health, and in recent years, it has emerged as an important factor leading to death (1). Recent studies have investigated the regulatory role of miRNAs in GC pathogenesis (2). Gastric cancer (GC) is one of the most prevalent cancers worldwide with a high rate of mortality (3). Risk factors of GC include helicobacter pylori infection, high age, diet low in vegetables, smoking, and a family history of GC (4, 5). Nevertheless, with improvement in GC therapy methods, survival rates of patients with advanced GC have remained low (6). Because GC is the second main leading cause of death in humans worldwide, it is necessary to introduce a suitable prognostic biomarker to improve survival rates in patients with GC. Currently, the diagnosis methods of GC are endoscopy and CT (7). Despite the invasiveness of the gastroscopy method, it is still the gold standard for diagnosis of early-stage gastric cancer. Therefore, about 30% of gastric cancers are diagnosed in the late stages, and the survival rate of patients with advanced stages is low (8, 9). 

Today, several biomarkers are used to diagnose or predict disease, including miRNAs associated with cancer development (10, 11). miRNAs are small non-coding RNAs that play a significant role in post-transcriptional gene regulation in several biological processes (12, 13). Accumulating evidence suggests that dysregulation of miRNAs contributes to the tumorigenesis, progression, and metastasis of different cancers, including GCs, in which the changed levels of distinct miRNAs provides diagnostic, prognostic, and predictive biomarkers of GCs (14). Evidence has shown that miRNAs deregulate in tumor cells and could be applied for tumor grading, detection, and prognosis. miRNAs play key roles in cancer cell proliferation, invasion, and apoptosis (15). The development mechanisms of GC are currently still unknown. In addition to genetic factors, miRNAs have recently been discovered to be one of the master-players in GC pathogenesis (16). Complementary to traditional diagnostic methods, identification of circulating miRNA biomarkers with high sensitivity is urgent (17). 

Several studies have reported differential expression profiles of miRNAs in various cancers including lung, breast, and other cancers. Recently, miRNAs have been identified as important contributors in molecular mechanisms of GC tumorigenesis and progression, which may participate in the development of new therapeutic strategies for GC patients (18). For example, the role of several miRNAs, including miR-506 (19), miR-616-3P (20), miR-422a (21), miR-181a (22), and others, in GC have been evaluated. Previous studies on miR-125 have indicated that miRNA expression levels are correlated with survival time in GC patients (23). Cai et al. suggested that miR-125a-5p might have potential prognostic value and also be potential therapeutic targets in gastric cancer (24). In their meta-analysis study, Zhang et al. suggested that miR-125a is a significant biomarker of prognosis in GC (25). Other previous study results have indicated that the miR-125a-3p is a potent prognostic marker in GC (26). The results of Dai et al. suggested that low expression of miR-125a predicts poor survival in GC patients (27). 

Accumulating reports have revealed the main role of the miR-125 family in the development of GC. Given the many contradictions in the prognostic value of the miR-125 family in GC patients, clarifying the prognostic role of this miRNA in GC will support the discovery of new therapeutic targets for GC. Therefore, in the present study, the data collected from studies was reassessed through a meta-analysis. 

## Methods

**Search strategy**

This meta-analysis was performed in accordance with the guidelines of the 2009 Preferred Reporting Items for Systematic Reviews and Meta-Analyses (PRISMA) statement (28). A systematic literature search of Google Scholar, Medline/PubMed, Embase, and ISI Web of Knowledge databases was performed to identify eligible studies published before December 2020 that reported comparative expressions of the miR-125 family between GC patients and healthy subjects. The following Mesh terms were used: (“gastric cancer” AND "microRNA-125a"), ("gastric cancer" AND "microRNA-125b), ("stomach cancer" AND "miR-125a), ("stomach cancer" AND "miR-125b"), (“gastric carcinoma” AND "microRNA-125a"), ("gastric carcinoma" AND "microRNA-125b), (“gastric neoplasm” AND "microRNA-125a"), ("GC" AND "microRNA-125b), (“GC” AND "microRNA-125a"), ("GC" AND "microRNA-125b). In addition, all references of retrieved publications were searched to identify relevant papers. Duplicate papers were removed from the analysis, and abstracts and full-text articles were reviewed by two independent reviewers (NA. D. and M.K.).

**Inclusion and exclusion criteria**

Inclusion criteria comprised: 1) publications reporting expression levels of miR-125 family and prognostic value in GC patients compared to control subjects; 2) assessments of expression levels of the miR-125 family in tissue or blood samples; 3) Studies with a retrospective design comprising 30 patients or more; and 4) Studies presenting sufficient information to estimate the HR and OR and corresponding 95% CI. 

Exclusion criteria were: 1) Studies that did not report diagnostic or prognostic value of miR-125 family in gastric cancer; 2) Studies that lacked valuable and quality data; and 3) Duplicate studies, letters, and review publications.

**Data collection**

Data extracted from each selected eligible paper comprised the first author’s name, publication year, country, study design, clinical stage of cancer, sample type, mean age of participants, clinicopathological features, miR-125 family, number of patients with gastric cancer, miR-125 family cutoff, method for assessing miR-125 family, follow-up time, outcome of prognosis, HR, 95% confidence interval, and NOS score of every included study. 

**Quality assessment**

Two independent investigators reviewed the full articles of all included studies and evaluated their quality based on the Newcastle–Ottawa scale (NOS) (29). The selection, comparability, and exposure of every included study were evaluated and assigned a score from 0-9. A NOS score <4 was considered a study with low quality, a score of 4 to 6 was determined as a moderate quality, and a NOS score ≥7 was considered a high quality study.

**Statistical analysis**

The pooled hazard ratio (HR) and odds ratio (OR) and their 95% CI were used to quantitatively determine the prognostic value of the miR-125 family in GC. In survival analyses, the hazard ratio (HR) is the ratio of hazard rates corresponding to the conditions described by two levels of an explanatory variable. An odds ratio (OR) is a measure of association between an exposure and an outcome. Heterogeneity among studies was assessed using the Q test and the Higgins I-square test (*p* <0.1, values <25%, 25%-50%, and >50% were set to indicate mild, moderate, and significant heterogeneity, respectively). The Begg’s rank correlation test and the Egger’s regression asymmetry test were also applied to evaluate any potential publication bias produced by the funnel plot (30, 31). Sensitivity analysis was also performed to evaluate the impact of removing studies with larger sample sizes on the overall effect size using the “leave-one-out method.” Meta-regression analysis based on the restricted maximum likelihood - random model was performed to evaluate the effects of confounder variables of lymph node metastasis, tumor progression, and liver metastasis on the prognosis of low expression of the miR-125 family in GC patients. All analyses were performed using CMA (comprehensive meta-analysis) V3 software (Biostat, NJ, USA) (32). A *p*-value threshold of 0.05 was used to determine statistical significance. 

## Results

**Comprehensive search of articles**

**Figure 1:**
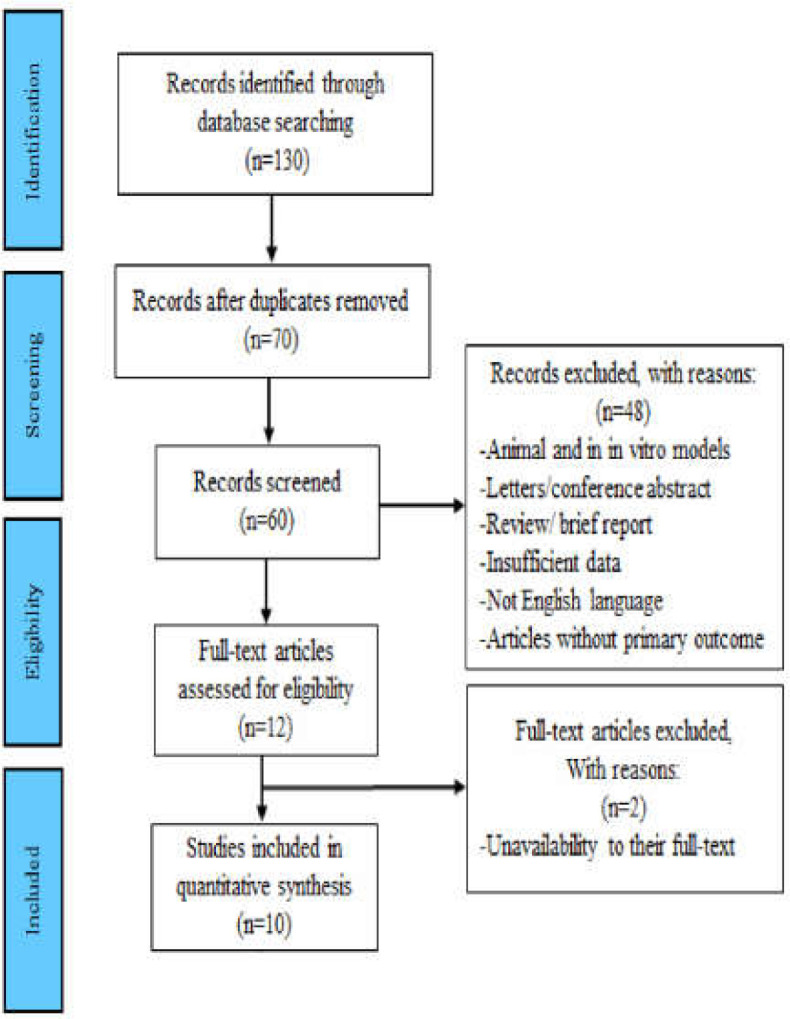
Flowchart detailing the selection of eligible studies for the meta-analysis

The literature search identified collectively 130 records in four databases associated with the topic of the current meta-analysis. Articles excluded were 48 duplicate articles, 18 reviews and brief reports, and 4 editorials from the primary search. The remaining 60 studies were further screened by title and abstract. Out of those 60 studies, 48 articles were excluded, because 4 articles had insufficient data; 3 studies were not in the English language; 10 articles were reported as brief reports, reviews, or letters; 18 articles did not report the circulating level of adiponectin as a primary outcome in GC in comparison with controls; and 11 articles were performed in animal and in vitro models. Two additional articles were excluded due to the unavailability of their full text. Finally, 10 articles were found to meet the inclusion criteria in the systematic and meta-analyses. Details of the study selection based on the PRISMA flowchart are shown in figure 1.

**Figure 2:**
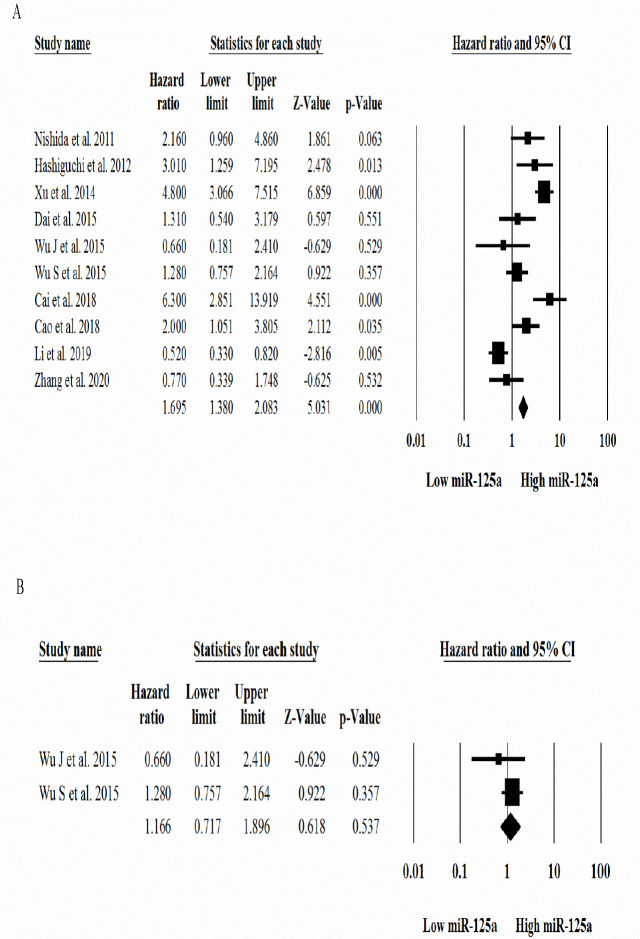
Forest plots of the prognostic value of the expression levels of A) miR-125a and B) miR-125b in patients with gastric cancer

**Table 1:** Key Characteristics of studies included in this meta-analysis

First author (year)	Country	Study design	Cancer stage	Sample type	MeanAge(year)	miR-125 family	Sample size	Assay method	Cutoff	Follow up (mo)	Outcome	HR	95% CI	NOS score
Nishida et al. 2011	Japan	Retrospective	I-IV	Frozen	67.2	125a	87	RT-PCR	None	147.6	OS (Univ)	2.16	(0.96, 4.86)	8
Hashiguchi et al. 2012	Japan	Retrospective	I-IV	Frozen	65	125a	70	RT-PCR	7.41	147.6	OS (Univ)	3.01	(1.26, 7.20)	8
Xu et al. 2014	China	Retrospective	I-IV	Frozen	65	125a	51	RT-PCR	None	-	-	4.8	(3.7, 8.9)	7
Dai et al. 2015	China	Retrospective	I-IV	FFPET	50	125a	73	RT-PCR	None	62	OS (Univ	1.31	(0.54, 3.18)	7
Wu J et al. 2015	China	Retrospective	I-IV	Frozen	67.1	125b	301	RT-PCR	4	60	OS (Univ)	0.66	(-0.96, 1.93)	8
Wu S et al. 2015	China	Retrospective	I-IV	Frozen	60	125b	73	RT-PCR	4	60	OS (Univ)	1.28	(-0.97, 4.6)	7
Cai et al. 2018	China	Retrospective	-	Frozen	65	125a	286	RT-PCR	None	90	OS (Univ)	6.3	(2.87, 14.01)	7
Cao et al. 2018	China	Retrospective	I-IV	Frozen	65	125a	82	RTPCR	None	80	OS (Univ)	2	(0.08, 8.1)	8
Li et al. 2019	China	Retrospective	I-IV	Frozen	60	125a	150	RTPCR	0.54	60	OS (Unive)	0.52	(0.33, 0.82)	7
Zhang et al. 2020	China	Retrospective	I-IV	Frozen	-	125a-5p	30	RTPCR	None	-	OS (Univ)	0.77	(-3.3, 7.6)	7

HR: hazard ratio, CI: confidence interval, NOS: Newcastle–Ottawa scale.

**Characteristics of accessible studies**

The baseline characteristics of the included studies were as follows: 1203 GC patients participated in this meta-analysis. In all studies, quantitative real-time PCR (qRT-PCR) was used to assess expression levels of the miR-125 family in frozen samples. Of these 10 studies, 8 and 2 studies were performed in China and Japan, respectively. These articles were published between the years 2011 and 2020. Studies by Nishida et al., Dai et al., Cai et al., Cao et al., Li et al. and Zhang et al. measured the expression levels of miR-125a-5p in GC patients in comparison with controls (24, 27, 33-36). Hashiguchi et al. estimated the expression of miR-125a-3p in patients with gastric cancer (26). Moreover, studies by Wu J et al., and Wu S et al. assessed the subclass of miR-125b in subjects with GC in comparison with controls (37, 38). In all of the studies, the source of miR-125 family was frozen tissue. Patients entered in this meta-analysis had a cancer stage between I and IV. The NOS score of all studies was ≥7. The main characteristics of the accessible studies are summarized in Table 1.

**Prognostic value of miR-125 family in GC**

The combined data of the included studies demonstrated a reduction in the expression levels of the miR-125 family in GC patients and indicated a strong association between low expression levels of the miR-125 family and susceptibility to GC as well as poor overall survival with HR calculated for miR-125a (HR = 1.7; 95% CI: 1.38 to 2.08; *p*< 0.001) (Figure. 2A) and miR-125b (HR = 1.16; 95% CI: 0.71 to 1.89; *p *= 0.53) (Figure. 2B). Sensitivity analysis was performed using the “leave-one-out method.” When the Wu J study with a large sample size was removed from among the included studies, the pooled effect size was not statistically significant (HR = 1.8; 95% CI: 0.99 to 3.4; *p *= 0.05). 

**Figure 3:**
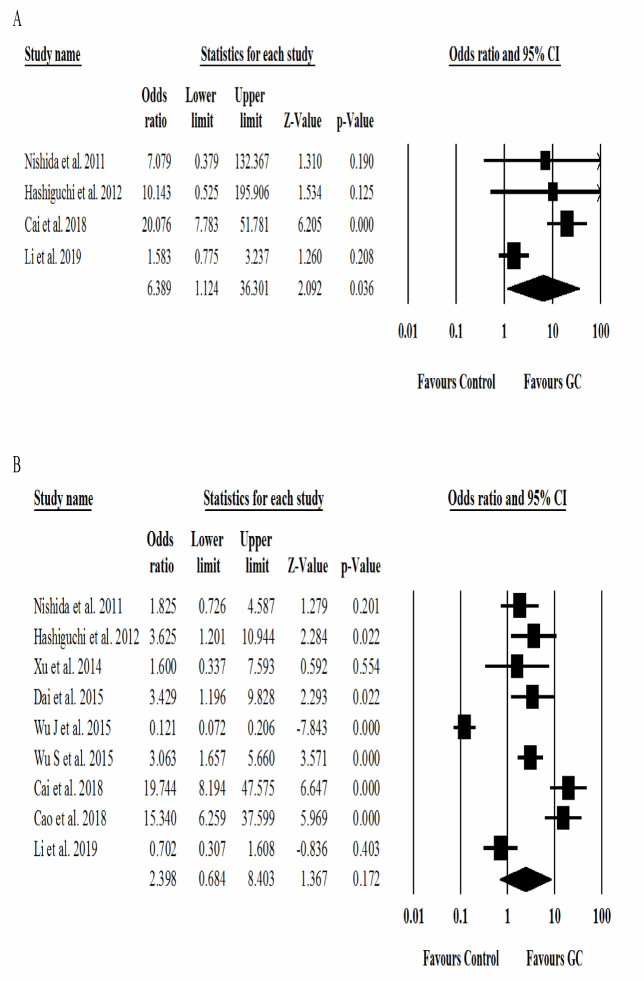
Forest plots of the association between the prognostic value of miR-125a with clinicopathological features of A) liver metastasis and B) lymph node metastasis

Removing each study in turn did not change the overall effect size that ranged between 1.70 and 1.84. In addition, a significant heterogeneity (I^2^ = 86%, *p*=0.00) was observed among studies with respect to demographic differences. 

**Correlation of miR-125 family expression with clinicopathological parameters**

Ten studies assessed odds ratio (OR) and 95% CI for the relationship between clinicopathological features such as liver metastasis, venous invasion, lymph node metastasis, and tumor progression. A random-effects model was applied to estimate effect size. The results revealed that liver metastasis (OR = 6.3; 95% CI: 1.1 to 36.3; *p *= 0.03) (Figure. 3A) along with low expression of the miR-125 family was significantly associated with an increased risk of GC. 

**Figure 4:**
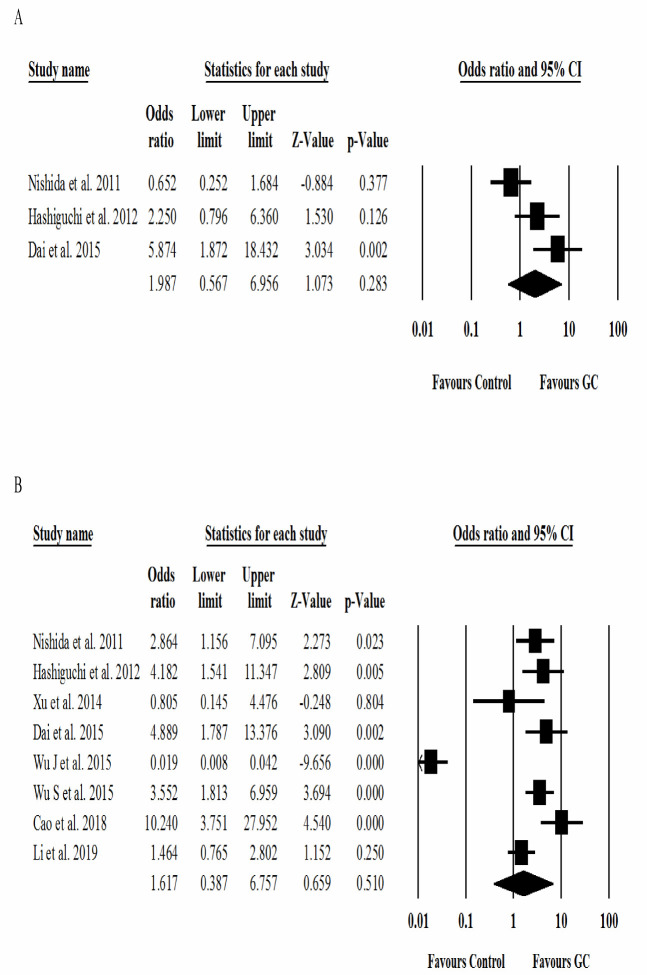
Forest plots of the association between the prognostic value of miR-125a with clinicopathological features of A) venous invasion and B) tumor progression

Other clinicopathological features such as lymph node metastasis (OR = 2.3; 95% CI: 0.68 to 8.4; *p *= 0.17) (Figure. 3B), venous invasion (OR = 1.9; 95% CI: 0.56 to 6.9; *p *= 0.28) (Figure. 4A), and tumor progression (OR = 1.6; 95% CI: 0.38 to 6.7; *p *= 0.51) (Figure. 4B) were not significantly correlated with increased risk of GC. However, the low expression of the miR-125 family and liver metastasis in GC patients may lead to poor overall survival and prolonged treatment time. 

**Publication bias**

**Figure 5:**
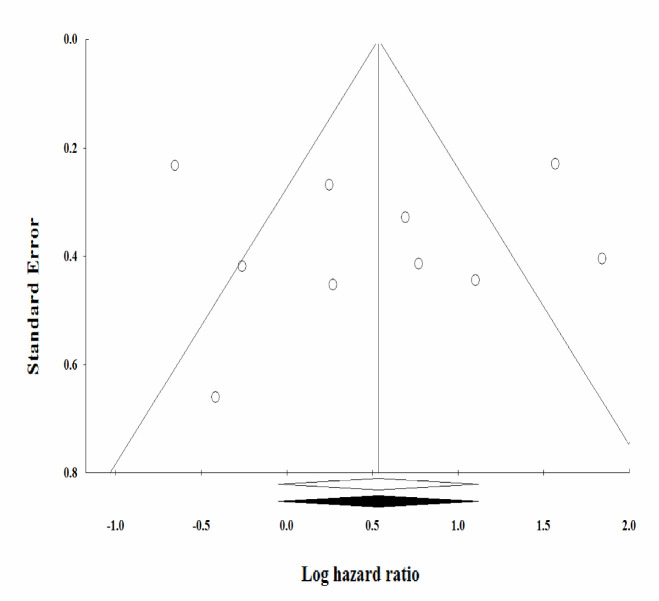
Funnel plot of log RR per standard error of identifying publication bias in the meta-analysis of the prognostic value of miR-125 family in patients with gastric cancer

The Begg's rank correlation and the Egger's regression asymmetry tests were used to assess the publication bias of all studies. The results of these tests (Begg's: *p* = 0.72 and Egger's: *p* = 0.95) were not significant. On the other hand, the funnel plot of the study precision by effect size was symmetric, suggesting no significant publication bias (Figure. 5). 

The observed publication bias was imputed using trim-and-fill correction. There were no imputed studies. 

**Figure 6:**
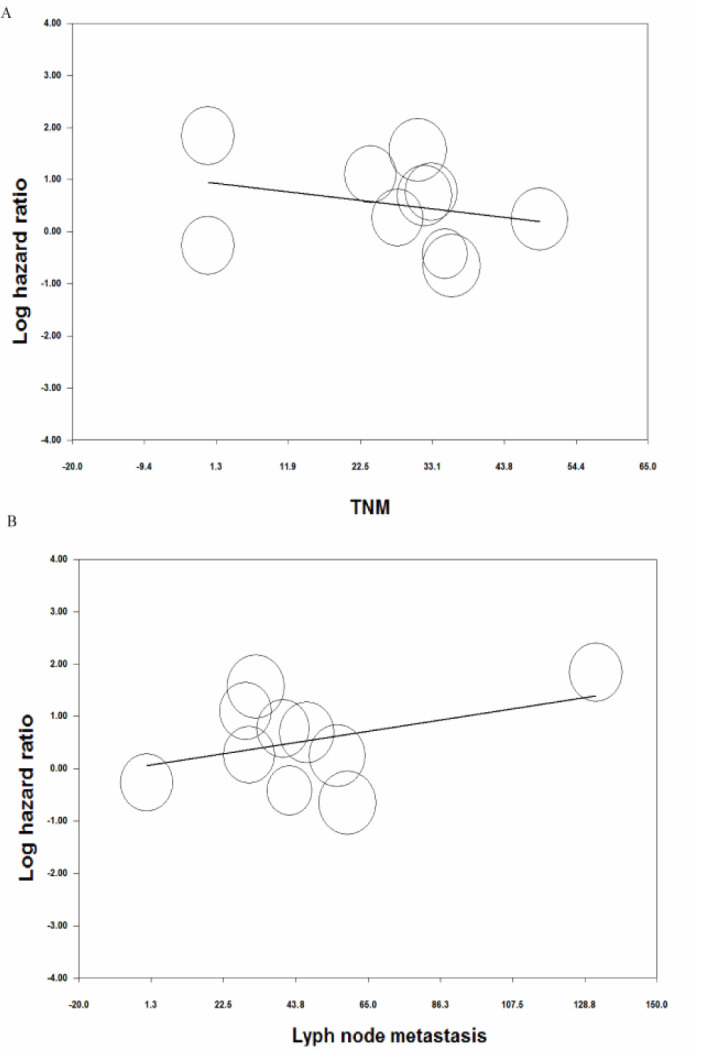
Meta-regression analysis for the effect of variables of A) TNM and B) lymph node metastasis on the prognostic value of miR-125a in patients with gastric cancer.

**Figure 7:**
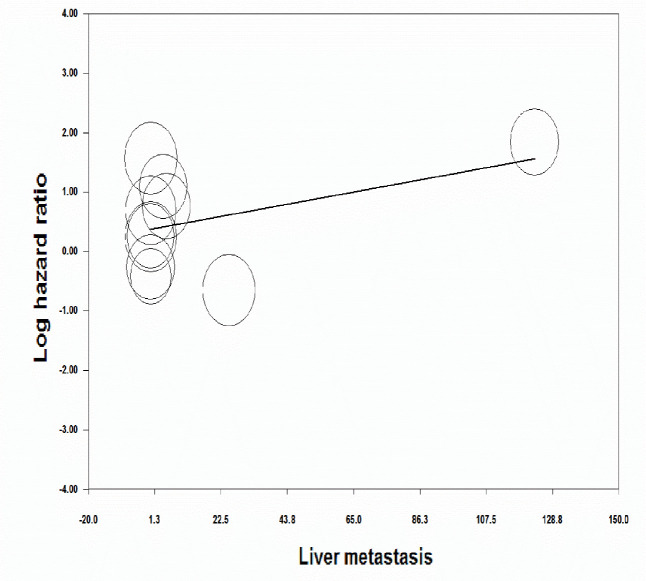
Meta-regression analysis for the effect of variable of liver metastasis on the prognostic value of miR-125a in patients with gastric cancer

**Subgroup analysis**

Based on the significant heterogeneity among studies, subgroup analysis was performed on subgroups of liver and lymph node metastasis, TNM stage, and sex. The results indicated no significant difference between the prognostic value of the miR-125a/b family and clinicopathological parameters. Moreover, sex had no impact on the prognostic value of miR-125a in patients with GC (data not shown). 

**Meta-regression analysis**

Meta-regression analysis was performed with respect to the TNM classification of GC and clinicopathological features including liver metastasis and lymph node metastasis to investigate inter-study heterogeneity. In the meta-regression analysis, it was found that the heterogeneity of results was not significantly influenced by the covariates of TNM classification (*p *= 0.40), lymph node metastasis (*[* = 0.22) (Figure. 6) or liver metastasis (*p *= 0.18) (Figure. 7). Therefore, the reason for the significant heterogeneity should have been searched in other factors among studies.

## Discussion

miRNAs are a member of small non-coding RNAs that regulate a wide array of biological processes including carcinogenesis (39). Recently, miRNAs have attracted increasing interest among researchers, especially for cancer investigations. A growing body of evidence suggests aberrantly expressed miR-125 family as a sign of malignant phenotype in GC (26, 27, 40). Among these studies, some have reported conflicting results on the prognostic role of the miR-125 family in GC patients. In the present study, meta-analysis was implemented to assess the prognostic value of the miR-125 family in GC patients. This study is the first meta-analysis to evaluate the prognostic and clinicopathological value of the miR-125a/b family, especially in gastric cancer. In the present study, pooled analysis of collected data from included studies through meta-analysis indicated that the low/high expression ratio of miR-125a is significantly associated with poor overall survival in GC patients. Nonetheless, the current results revealed that the expression of miR-125b was not related to the prognosis of survival rate in GC.

miR-125 is a family of microRNAs that are involved in various cancer types (41). Several previous studies have shown an obvious role of miR-125 in the proliferation, migration, and invasion of different cancers (42, 43). In addition, miR-125a is related to tumor cell growth, differentiation, and metastasis (24, 44). Apart from that, miR-125a regulates the MEK1/2/ERK1/2 signaling pathway in different cell types (45). It also has a regulatory role in the function of EST gene, vascular endothelial growth factor A (VEGF-A), ErbB2 and ErbB3 signaling pathways, and ILR (40). Taken together, the current findings support the prognostic value of miR-125a in tissue-based samples of gastric cancer. This could be due to plenty of miRNAs in tissue samples in contrast to blood samples. According to a study reported in 2019, the prognostic value of miR-125a was not seen in blood samples (46). 

In the next step, a significant heterogeneity was observed among studies. To explore the source of heterogeneity, subgroup analysis was performed based on the subgroups of liver and lymph node metastasis, TNM stage, and sex. At first, the prognostic value of miR-125a was estimated in subgroups of patients with or without liver and lymph node metastasis. The results indicated no significant difference in the prognostic value of miR-125a in patients with liver and lymph node metastasis compared to patients it. Furthermore, the prognostic value of miR-125a was analyzed in two subgroups of TNM stages I and II and TNM stages III and IV in GC patients. The pooled analysis for the two subgroups showed no significant difference in the prognostic value of miR-125a. Moreover, it was found that sex has no effect on the prognostic value of miR-125a in patients with GC. These results were confirmed by meta-regression analysis. Meta-regression analysis showed no significant association between the prognostic value of miR-125a and confounder variables of liver and lymph node metastasis and TNM stage in GC patients. Taken together, the present results confirm that the relationship between low miR-125a expression and poor survival rate is not dependent upon clinicopathological features. 

This study had several limitations. One was the significant heterogeneity among included studies. To explore the source of heterogeneity, subgroup analysis was performed. The relatively small sample size of primary studies involved in the subgroup analysis could be another limitation of the present meta-analysis. However, to validate these results, further studies are required with larger sample sizes. 

The present meta-analysis found that low miR-125a expression could be a biomarker in the prognosis of poor survival rate in GC patients. However, with respect to the small sample size, the results should be interpreted cautiously.

## Conflict of interests

The authors declare that they have no conflict of interest.
